# Emergency department visits due to severe falls: comparing patient self- reports and general practitioner records: A cross-sectional study

**DOI:** 10.1186/s12877-025-06411-9

**Published:** 2025-10-06

**Authors:** Greta Fellhölter, Tim Stuckenschneider, Laura Himmelmann, Tania Zieschang

**Affiliations:** Department for Health Services Research, Geriatric Medicine, School of Medicine and Health Services, Carl von Ossietzky University, Ammerländer Heerstraße 114-118, Lower Saxony, 26129 Oldenburg, Germany

**Keywords:** Older people, Self-report, Morbidity, Emergency department, Falls prevention

## Abstract

**Background:**

Older individuals who experience a severe fall are at high risk for long-term consequences and require structured follow-up care including risk assessments and secondary prevention (e.g. exercise). Accurate knowledge of pre-existing diagnoses is essential for tailoring interventions. However, identifying these diagnoses in emergency department (ED) is challenging due to time constraints, high workload, potential recall bias after a traumatic event, and limited electronic data exchange across healthcare settings in Germany. This study analyses the concordance between self-reported diagnoses of adults aged > 60 years presenting to the ED after a fall without hospitalisation and diagnostic information provided by their general practitioners (GPs).

**Methods:**

Data from the SeFallED study conducted in Germany were analysed. To analyse concordance, 28 major diagnostic groups (e.g. heart diseases, cancer, lung diseases) were established. Cohen’s Kappa assessed the agreement between self-reported and GP-reported diagnoses. Logistic regression identified associations between population characteristics (e.g., cognition, concerns about falling, age) and discordance between self-reported and GP-reported diagnoses.

**Results:**

A total of 216 participants (mean age 75.2), with an average of five diagnoses per person, were included. Agreement was almost perfect (K = 0.81-1.0) for Parkinson’s disease, substantial (K = 0.61–0.80) for diabetes mellitus and cancer, and moderate (K = 0.41–0.60) for heart and lung diseases. Other conditions showed fair, poor, or no agreement. Age, sex, BMI, cognition, concerns about falling, education, and living arrangements were associated with discordance. Only higher concerns about falling were linked to non-agreement for overall diagnoses.

**Conclusion:**

Overall, there was poor agreement between self-reported and GP-reported diagnoses. Special attention should be given to older adults and individuals with high concerns about falling, as these factors predicted discordance. Participants were more likely to report diseases with more noticeable symptoms and frequent monitoring such as Parkinson’s disease or diabetes mellitus. To optimise follow-up care, improving the accuracy of diagnostic information is essential, which may be facilitated by harmonizing patient information across electronic systems (e.g., electronic health cards). Improved communication between GPs and patients regarding existing illnesses is also crucial to enhance the accuracy of patient self-reports.

**Trial registration:**

DRKS (Deutsches Register für klinische Studien, DRKS00025949, prospectively registered on 4th November, 2021).

**Supplementary Information:**

The online version contains supplementary material available at 10.1186/s12877-025-06411-9.

## Background

Falls are a significant health hazard in older adults, with incidence and mortality rates increasing with advancing age [1]. Annually, 28–35% of individuals aged 65 years and older experience a fall, which emphasises the urgency of targeted prevention and intervention strategies such as medication review and structured exercise [2, 3]. These measures are part of the structured follow-up care recommended by the world guidelines for falls prevention for individuals who have suffered severe falls, which identifies them as having a high risk of future functional decline. According to the guidelines severe falls are characterised by the necessity of ED visits due to injuries, an inability to get up unassisted for at least an hour following a fall and an association with loss of consciousness [3]. To date, structured follow-up is not part of emergency department (ED) care, which primarily focuses on acute treatment rather than addressing the causes or consequences of a fall. However, identifying and managing underlying causes is crucial to prevent recurrent falls and their associated consequences.

Given their increased vulnerability to serious fall-related consequences, older people classified at “high risk” require a tailored follow-up plan including a comprehensive multifactorial assessment addressing concerns about falling and potential cognitive impairment [3]. Age-related diseases such as osteoporosis can significantly influence the long-term sequelae following a fall, increasing the likelihood of severe outcomes, such as fractures, reduced mobility, and heightened mortality [4, 5]. Therefore, an accurate and thorough medical history is crucial to optimise the treatment and follow-up of older individuals after a severe fall. Currently, diagnoses are typically obtained through patient self-report at ED presentation, as data linkage between healthcare settings – such as GPs and emergency care – is not yet routinely established. Automated electronic exchange of patient information remains unavailable and requires patient consent for data sharing across systems. However, previous studies have indicated that self-reported medical histories in older individuals may be unreliable [6, 7]. Factors such as higher age, female sex and lower education level have been identified to affect the accuracy of self-reports [6, 7].

To date, no research has specifically investigated the factors that affect the agreement between self-reported diagnoses and those reported by general practitioners (GPs) in older adults, who have experienced a severe fall with presentation to the ED. Given the potential long-term consequences these individuals may face and the possible impact of preexisting conditions, it is essential to identify characteristics that may necessitate proxy assessments in addition to self-assessments. While obtaining medical information from proxies or GPs can offer valuable insights, it also demands additional resources, particularly the time of healthcare professionals, which may come at the expense of direct patient interaction. Thus, this study aims to evaluate the added value of these sources and identify associations of demographic factors with the accuracy of self-reported medical histories. By critically assessing the informational value of different data sources (i.e., GP reports and self-reports), this research seeks to enhance post-fall care and develop personalised treatment plans as integral components of falls prevention strategies. Furthermore, this study investigates the potential associations of age- and fall-specific screening tools, such as the Falls Efficacy Scale-International (FES-I) and the Montreal Cognitive Assessment (MoCA), both which are recommended in the world guidelines for falls prevention [3].

## Methods

### Study population

Participants were recruited from the SeFallED-Study (Sentinel Fall presenting to the Emergency Department), which is a mixed-methods study conducted at the Carl von Ossietzky University in Oldenburg, Germany [8]. The SeFallED-Study examines long-term trajectories of older adults (aged 60 years and older) who present to the ED following a severe fall without subsequent hospital admission [8]. The recruitment period spanned from November 2021 to December 2023. Further information on the recruitment processes can be found in the corresponding paper [9]. The SeFallED-Study adheres to the principles outlined in the Declaration of Helsinki (1974) and its subsequent amendments [8]. It has been prospectively registered in the German Clinical Trials Register (DRKS-ID: 00025949) and received approval from the Medical Ethics Committee of the University Oldenburg (number 2021 − 120) [8]. Baseline data from the observational prospective study were used for this sub-study. Participants were eligible if they met the following criteria: (1) aged 60 and above, (2) presentation to the Emergency Department (ED) of either the Klinikum Oldenburg or the Evangelische Krankenhaus Oldenburg with a fall without subsequent hospitalisation, (3) written informed consent [8]. The exclusion criteria include a life expectancy of less than three months, unstable medical, neurological, or psychiatric conditions, being bedridden or unable to walk without support from another person, residing more than 40 km from the research centre, acute psychosis or social aggression, and an inability to communicate verbally in German or English [8]. For this sub-study, participants or their caregivers were required to provide an additional signature consenting to contact their GP and waive medical confidentially obligation, given the heightened protection of medical information. This additional consent was optional and voluntary for participants in the SeFallED-Study.

### Data collection

#### Participants characteristics

An extensive geriatric assessment was conducted at baseline of the SeFallED study, which was performed at the participants’ home. It included assessing cognitive function using the MoCA [10] as well as analysing concerns about falling using the short FES-I [11, 12]. The MoCA ranges between 0 and 30, with higher points indicating better cognitive performance [10, 13]. The short version of the FES-I is a validated and practical tool comprising seven items related to daily activities [12]. Participants rate their concern about falling during each activity on a 4-point scale (1 = not at all concerned to 4 = very concerned [11, 12]. The total score ranges from 7 to 28 points, with higher scores indicating greater concern about falling [12]. Scores of 7–8 indicate low concern, 9–13 moderate concern and 14–28 high concern [14].

Besides assessing cognitive performance and concerns about falling, sex (male, female), age (years), BMI, education (low (< 10 years), middle (10–12 years), high (> 12 years)) and living arrangement (alone, with others, institution, others) were assessed. For BMI, individuals’ height and weight was taken using a mobile scale and stadiometer. Further data was assessed via interview, in case of severe cognitive impairment via a proxy (e.g., family member or legally authorised representative).

#### Self-reported diagnoses

As part of the baseline assessment, participants were asked to identify all preexisting comorbidities, which were documented by the study-team. Self-reported diagnoses, often provided in lay language by participants, were later assigned an ICD-10 (International Classification of Disease 10) code by the study team. The ICD-10 is a statistical classification system that captures diseases and sorts them by categories and groups to facilitate the analysis of morbidity rates on an international level [15]. Names of diseases are translated in a code including numbers and letters [15]. Data was stored in Red Cap hosted at the Carl von Ossietzky University Oldenburg. Red Cap is a secure platform managing data for research studies [16].

#### Reported diagnoses from GP

The GP was contacted with a request for the current medication plan and diagnoses if informed consent has been given. A stamped envelope was provided for the return shipment of medical records. All documents were pseudonymised, scanned, and then electronically stored on specific data protected servers of the university. One researcher viewed all documents and extracted all diagnoses noted into an excel spreadsheet for further analyses.

### Data Preparation

Consistent with previous research that utilised various diagnostic groups to categorise self-reported conditions such as diabetes mellitus, cancer, or asthma [7, 17], we established primary diagnostic groups. A researcher initially identified common diagnoses from medical records, which were then reviewed in consultation with medical professionals. This process resulted in the formation of 28 major diagnostic groups. Analysed diagnostic groups can be found in Table 1. Diagnoses characterised as acute with no lasting effects, meaning they did not become chronic (e.g. flu-like infection, cystitis), were excluded. Additionally, diagnoses that do not significantly impact physical performance or susceptibility to falls (e.g. seborrheic keratosis) were also excluded, as well as diseases that occurred in fewer than 10 participants simultaneously in self-reports and GP-reports. In line with the recommendations of the world guidelines for falls prevention, which emphasise the importance of considering diseases associated with increased fall risk (e.g. Parkinson`s Diseases (PD) [3], diagnoses that are particularly related to falls are included in the analysis, even if they are reported by fewer than 10 individuals. This procedure was established by consensus within the geriatric team, in conjunction with a review by medical professionals and based on previous studies, which also primarily assessed chronic conditions [6, 7, 17].

**Table 1:** Diagnostic groups and included diagnoses

Diagnostic groups		Included diagnoses
Heart diseases		Arterial hypertension, and fibrillation, extrasystole, cardiac arrhythmia, tachycardia, bradycardia, palpitations, right and left bundle branch block, atrioventricular block, heart failure, hypertensive heart disease, coronary heart disease, stent implantation, pacemaker
PAD (Peripheral Arterial Disease)		PAD
Stroke (apoplexy)		Carotid stenosis, stenosis of arteria cerebri media, cerebral microangiopathy, vascular encephalopathy, arterial cerebral sclerosis, not further specified cerebrovascular disease, stroke, anamnestic stroke, consequences of stroke, subclavian steal syndrome, transient ischemic attack
Cancer		Carcinomas of any kind
Lung diseases		Bronchial asthma, allergic asthma, chronic bronchitis, chronic obstructive pulmonary disease (COPD), bronchiectasis
Gastrointestinal diseases		Gastroesophageal reflux disease (GERD), Barrett`s esophagus, gastritis, ileus/coprostasis, obstipation/defecation disorder, irritable bowel syndrome, diverticulosis, cholecystolithiasis, choledocholithiasis and others, cholestasis, dysphagia
Renal diseases		Renal insufficiency, other chronic renal disease and diseases concerning the ureter
Incontinence		Urinary and faecal incontinence, miction disorder, overactive bladder
Ophthalmic diseases		Presbyopia, astigmatism, hyperopia, optic atrophy, amblyopia, glaucoma, cataract, Fuchs corneal dystrophy, fundus hypertension, vitreous detachment, implants
Diabetes mellitus		All types of diabetes mellitus with and without complications
Polyneuropathy		All types of polyneuropathies, e.g. alcohol polyneuropathy
Cognitive impairment		Cognitive disorder, intelligence reduction, general forgetfulness, dementia, Alzheimer`s disease, senility
PD (Parkinson`s disease)		PD
Gait disorders		Gait disorders
Frailty		Frailty
Risk of falling		Risk of falling
Depression		All types of depression, psychosomatic disorder, psychovegetative exhaustion
Sleeping disorders		Sleeping disorders, sleep apnea
Pain		Chronic pain, chronic pain disorder with somatic and mental components, rheumatism, pain of the extremities, fibromyalgia
Vertigo		Vertigo, benign paroxysmal positional vertigo, syncope
Tremor		Tremor, Restless Legs Syndrome
Osteoporosis		Osteoporosis
Orthopedic diseases	Upper extremities	Cartilage diseases, Different lengths of extremities, Pelvic obliquity, Chronic instability of the knee joint and the ankle, Syndromes of the cervical, thoracic, and lumbar vertebrae, Spondylosis, Spondylopathy, Spinal disc herniation, Implants, Impingement syndrome of the shoulder, Periarthropathia humeroscapularis
	Lower extremities	Cartilage diseases, Different lengths of extremities, Pelvic obliquity, Chronic instability of the knee joint and the ankle, Syndromes of the cervical, thoracic, and lumbar vertebrae, Spondylosis, Spondylopathy, Spinal disc herniation, Implants, Sinking splayfoot, Static foot complaints, Leg shortening, Meniscus lesion
Arthrosis	Upper extremities	General arthrosis, polyarthritis, arthrosis of upper and lower extremities, chronic post-rheumatic arthrosis, general arthritis, polyarthritis, psoriasis- associated arthritis, arthritis of upper and lower extremities
	Lower extremities	General arthrosis, polyarthritis, arthrosis of upper and lower extremities, chronic post-rheumatic arthrosis, general arthritis, polyarthritis, psoriasis- associated arthritis, arthritis of upper and lower extremities

### Statistical analysis

To assess whether the subsample was representative of the larger study population, we conducted group comparisons using independent t-tests for normally distributed variables and Mann-Whitney U tests for non-normally distributed variables. Chi-square tests were used for the dichotomous variable sex.

Each self-reported diagnosis was matched to the corresponding diagnosis provided by the GP. To evaluate agreement between self- and GP-reported diagnoses, Cohen’s Kappa was calculated. Interpretation followed standard thresholds: 0.81-1.0 = almost perfect, 0.61–0.80 = substantial, 0.41–0.6 = moderate, 0.21–0.4 = fair, 0.01–0.2 = poor [6, 7, 18, 19].

To identify associations with overall diagnostic agreement (dependent variable), we calculated an individual agreement score per participant across all diagnostic categories. Multivariate logistic regression was used to examine associations with patient characteristics (independent variables), reporting odds ratios (OR) and 95% confidence intervals (CI).

Additionally, we performed separate multivariate logistic regression analyses for each of 27 diagnostic categories to explore factors associated with binary agreement (outcome variable: yes/no) between self- and GP-reported diagnoses. Parkinson’s disease was excluded due to insufficient case numbers. Independent variables in all models included age, sex, BMI, MoCA score, short FES-I score, education level, and living arrangement.

No a priori power calculation was performed, as this was an exploratory analysis using a subsample from a larger prospective study. Multicollinearity among the independent variables was assessed using a linear regression; all variance inflation factors (VIFs) [20] were below 1.4, indicating minimal multicollinearity. Statistical Analyses were conducted by using IBM SPSS Statistics, Version 29.0.0.

## Results

### Participant characteristics

Of the 335 participants initially enrolled in the SeFallED study, 40 did not provide written consent to contact their general practitioner (GP), and medical records were not returned by 79 GPs. This resulted in a final analytical sample of 216 participants. The mean age of included participants was 75 years. On average, they reported moderate concerns about falling; no participant reached the maximum short FES-I score of 28. The mean MoCA score was 23. Characteristics of the full SeFallED cohort (*n* = 335) and the analysed subset (*n* = 216) are presented in Table 2. Statistical comparisons showed no significant differences between the two groups, indicating that the analysed sample is representative of the full cohort. On average, GPs reported 4.6 (± 3.1) diagnoses per patient.

**Table 2:** Characteristics of the study participants

	SeFallED cohort *n* = 335	SeFallED subset *n* = 216	*p*-value
Age, years (mean, CI)	75.4 (74.5–76.4)	75.2 (74.0-76.5)	*0.762*
Sex			*0.473*
female, *n* (%)	211 (63.0)	129 (59.7)	
male, *n* (%)	124 (37.0)	87 (40.3)	
BMI (mean ± SD)	27.4 ((± 5.4)	27.6 (± 5.5)	*0.836*
MoCA	23.2 (± 4.1)	23.2 (± 4.2)	*0.902*
Short FES-I	10.5 (± 4.6)	10.4 (± 4.5)	*0.953*
Education			*0.800*
low (< 10 years), *n* (%)	158 (47.2)	108 (50.0)	
middle (10–12 years), *n* (%)	100 (29.9)	62 (28.7)	
high (> 12 years), *n* (%)	77 (23.0)	46 (21.3)	
Living arrangement			*0.981*
alone, *n* (%)	121 (36.1)	74 (34.3)	
with others, *n* (%)	198 (59.1)	128 (59.3)	
institution, *n* (%)	15 (4.5)	13 (6.0)	
other, *n* (%)	1 (0.3)	1 (0.5)	

If not otherwise stated data is presented in n (%) or mean (± SD), *BMI* body mass index, *MoCA* Montreal Cognitive Assessment, *Short FES-I* Short Falls Efficacy Scale

### Agreement between Self-Reported and GP-Reported diagnoses

The prevalence of diagnostic groups and corresponding Cohen’s Kappa values are presented in Table 3, illustrating agreement between self-reported and GP-reported diagnoses. ICD-10 codes of corresponding diagnoses are included in Supplementary Table S1. The most commonly self-reported diagnoses included heart diseases, general arthrosis, ophthalmic diseases, diabetes mellitus, general orthopaedic diseases and cancer. GPs most frequently reported heart diseases and general orthopaedic diseases followed by general arthrosis, gastrointestinal diseases and diabetes mellitus.

**Table 3:** Agreement between self-reported diagnoses and GP reported diagnoses

Diagnostic groups	Self reported		GP reported			
	n	%	n	%	Kappa	SD
Heart diseases	120	55.6	157	72.7	0.503	0.06
PAD	4	1.9	45	20.8	0.092	0.06
Stroke	12	5.6	40	18.5	0.411	0.08
Cancer	30	13.9	40	18.5	0.762	0.06
Lung disease	16	7.4	42	19.4	0.459	0.08
Gastrointestinal diseases	23	10.6	77	35.6	0.282	0.06
Renal diseases	6	2.8	37	17.1	0.096	0.07
Incontinence	3	1.4	10	9.3	0.064	0.08
Ophthalmic diseases	38	17.6	23	10.6	0.074	0.07
Diabetes mellitus	32	14.8	52	24.1	0.679	0.06
Polyneuropathy	6	2.8	29	13.4	0.311	0.10
Cognitive disorders	2	0.9	16	7.4	0.096	0.10
PD	3	1.4	3	1.4	1.000	0.00
Gait disorders	1	0.5	26	12	−0.01	0.01
Frailty	0	0	36	16.7	-	-
Risk of falling	0	0	17	7.9	-	-
Depression	7	3.2	31	14.4	0.166	0.09
Sleeping disorders	1	0.5	19	8.8	0.092	0.09
Pain	1	0.5	31	14.4	0.054	0.05
Vertigo	1	0.5	38	17.6	0.043	0.04
Tremor	2	0.9	12	5.6	0.274	0.15
Osteoporosis	20	9.3	31	14.4	0.359	0.09
General orthopedic diseases	31	14.4	122	56.5	0.178	0.04
Orthopedic diseases upper extremities	2	0.9	20	9.3	0.168	0.10
Orthopedic diseases lower extremities	6	2.8	60	27.8	0.030	0.04
General arthrosis	39	18.1	91	42.1	0.125	0.06
Arthrosis lower extremities	6	2.8	69	31.9	0.002	0.03
Arthrosis upper extremities	3	1.4	28	13.0	0.040	0.06

If not otherwise stated, data is presented in *n*, % Cohen’s Kappa and SD; *PD* Parkinson’s disease, *PAD* peripheral arterial disease

Agreement between data sources varied across diagnoses. Perfect agreement was observed for PD. Substantial agreement was found for diabetes mellitus and cancer. Moderate agreement was observed for heart diseases, stroke and lung diseases, while agreement was fair for osteoporosis, polyneuropathy, gastrointestinal diseases and tremor. Poor agreement was noted for all other diagnostic groups. No agreement was found for gait disorders and arthrosis of the lower extremities. Cohen’s Kappa could not be calculated for frailty and risk of falling, as these were exclusively reported by GPs, indicating complete discordance.

### Associations between diagnostic agreement and patient characteristics

Table 4 presents the association between participant characteristics and disagreement between self-reported and GP-reported diagnoses. Overall diagnostic agreement (i.e., across all diagnoses) was significantly associated with the FES-I score. For each additional point on the FES-I, the odds of disagreement increased by 1.86 times (95% CI 1.08, 3.19).

**Table 4:** Association between characteristics of the study population and disagreement between self-reported and GP-reported diagnoses

	**Overall**	**Heart diseases**	**PAD**	**Stroke**	**Cancer**	**Lung diseases**	**Gastrointestinal diseases**	**Renal diseases**
Age	1.08 (1.00-1.18)	1.01 (0.97-1.06)	1.05 (1.00-1.10)*	1.10 (1.03-1.18)*	1.11 (1.01-1.21)*	1.04 (0.98-1.10)	1.01 (0.97-1.06)	1.08 (1.02-1.14)*
Sex								
male								
female	2.56 (0.78-8.45)	2.00 (0.92-4.33)	0.79 (0.38-1.63)	1.07 (0.41-2.77)	0.75 (0.21-2.72)	0.70 (0.28-1.71)	0.86 (0.43-1.73)	0.86 (0.37-2.00)
BMI	1.07 (0.93-1.22)	0.92 (0.85-0.99)*	1.03 (0.96-1.10)	0.96 (0.87-1.05)	0.99 (0.86-1.15)	1.06 (0.99-1.15)	1.08 (1.02-1.15)*	1.04 (0.97-1.12)
MoCA	0.96 (0.77- 1.20)	0.96 (0.86-1.07)	1.05 (0.93-1.18)	0.99 (0.87-1.14)	1.29 (1.01-1.64)*	0.99 (0.87-1.12)	0.96 (0.87-1.06)	1.04 (0.92-1.17)
FES-I	1.86 (1.08-3.19)*	1.14 (1.05-1.25)*	0.95 (0.86-1.05)	0.99 (0.89-1.10)	0.95 (0.78-1.15)	1.01 (0.92-1.12)	1.07 (0.99-1.16)	1.10 (1.01-1.20)
Education								
low								
medium	3.98 (0.85-18.60)	3.37 (1.47-7.94)*	0.73 (0.30-1.73)	0.83 (0.27-2.61)	0.50 (0.09-2.71)	0.62 (0.20-1.91)	0.76 (0.34-1.73)	1.51 (0.54-4.20)*
high	2.69 (0.63-11.46)	2.31 (0.85-6.25)	0.70 (0.27-1.83)	1.02 (0.30-3.41)	0.47 (0.09-2.57)	0.85 (0.26-2.80)	0.94 (0.37-2.34)	3.47 (1.22-9.85)
Living arrangement								
alone								
together	/#	2.19 (0.98-4.91)	0.92 (0.43-1.98)	0.98 (0.36-2.66)	/#	0.85 (0.32-2.22)	0.69 (0.34-1.41)	0.85 (0.36-2.03)
institution	/#	0.81 (0.14-4.70)	0.30 (0.34-2.69)	1.64 (0.33-8.29)	/#	1.77 (0.35-8.99)	0.27 (0.05-1.59)	0.44 (0.07-2.874)
	**Incontinence**	**Ophthalmic diseases**	**Diabetes mellitus**	**Polyneuropathy**	**Cognitive disorders**	**Gait disorders**	**Frailty**	**Risk of falling**
Age	1.03 (0.97-1.10)	1.03 (0.99-1.08)	1.06 (0.99-1.13)	1.05 (0.99-1.12)	1.06 (0.98-1.16)	1.18 (1.08-1.29)*	1.18 (1.09-1.27)*	1.07 (0.98-1.16)
Sex								
male								
female	1.52 (0.52-4.48)	0.88 (0.43-1.81)	1.88 (0.65-5.46)	0.36 (0.13-0.96)*	0.50 (0.15-1.68)	0.44 (0.14-1.38)	0.82 (0.33-2.08)	2.18 (0.56-8.48)
BMI	1.04 (0.96-1.13)	0.98 (0.92-1.05)	1.02 (0.94-1.11)	1.13 (1.04-1.23)*	1.06 (0.96-1.19)	1.03 (0.92-1.15)	1.02 (0.93-1.11)	0.90 (0.80-1.02)
MoCA	0.98 (0.86-1.12)	0.98 (0.86-1.12)	1.01 (0.88-1.16)	1.13 (0.97-1.31)	0.89 (0.76-1.04)	1.16 (0.98-1.37)	0.97 (0.85-1.10)	0.84 (0.70-0.99)*
FES-I	1.08 (0.98-1.20)	1.08 (0.98-1.20)	1.12 (1.02-1.24)*	1.04 (0.92-1.16)	1.06 (0.94-1.18)	1.04 (0.93-1.17)	1.01 (0.92-1.12)	1.05 (0.93-1.19)
Education								
low								
medium	1.05 (0.32-3.47)	1.05 (0.32-3.47)	1.07 (0.32-3.61)	0.54 (0.16-1.82)	0.47 (0.09-2.43)	0.67 (0.18-2.50)	0.57 (0.18-1.80)	0.79 (0.15-4.02)
high	0.96 (0.22-4.15)	0.96 (0.22-4.15)	1.80 (0.49-6.65)	0.52 (0.14-1.96)	0.38 (0.04-3.42)	0.22 (0.03-1.51)	0.58 (0.16-2.12)	0.94 (0.15-5.73)
Living arrangement								
alone								
together	1.02 (0.35-3.02)	1.02 (0.35-3.02)	2.38 (0.74-7.68)	0.48 (0.17-1.35)	/#	0.76 (0.22-2.58)	1.24 (0.46-3.33)	1.63 (0.39-6.78)
institution	1.34 (0.21-8.39)	1.34 (0.21-8.39)	2.93 (0.51-16.77)	0.34 (0.03-3.60)	/#	2.84 (0.54-15.09)	0.44 (0.07-2.68)	8.63 (1.39-53.84)*
	**Depression**	**Sleep disorders**	**Pain**	**Vertigo**	**Tremor/RLS**	**Osteoporosis**	**General orthopedic diseases**	**Orthopedic diseases - upper extremities**
Age	0.97 (0.91-1.02)	0.97 (0.91-1.04)	1.02 (0.97-1.08)	1.04 (0.99-1.10)	1.04 (0.95-1.14)	1.04 (0.98 -1.11)	0.99 (0.95-1.03)	1.01 (0.94-1.08)
Sex								
male								
female	1.24 (0.47-3.24)	0.52 (0.18-1.46)	2.41 (0.89-6.53)	0.90 (0.39-2.08)	0.87 (0.22-3.45)	3.65 (1.15-11.57)*	1.14 (0.62-2.09)	1.01 (0.34-2.30)
BMI	0.93 (0.86-1.02)	1.06 (0.98-1.16)	1.05 (0.97-1.13)	0.99 (0.92-1.07)	1.00 (0.87-1.14)	0.97 (0.89-1.06)	1.09 (1.03-1.16)*	1.01 (0.91-1.11)
MoCA	1.11 (0.96-1.27)	0.99 (0.85-1.16)	1.02 (0.90-1.15)	0.97 (0.86-1.09)	0.96 (0.78-1.19)	0.98 (0.86-1.12)	0.98 (0.89-1.08)	1.11 (0.93-1.32)
FES-I	1.17 (1.06-1.29)*	1.00 (0.88-1.16)	1.03 (0.93-1.14)	1.09 (1.00-1.19)*	0.95 (0.79-1.14)	0.96 (0.85-1.08)	0.97 (0.90-1.05)	0.97 (0.84-1.12)
Education								
low								
medium	0.59 (0.20-1.74)	0.37 (0.09-1.56)	0.69 (0.25-1.89)	2.69 (1.05-6.90)*	0.34 (0.04-3.03)	1.17 (0.43-3.20)	0.61 (0.30-1.24)	0.48 (0.14-1.72)
high	0.52 (0.15-1.80)	0.73 (0.19-2.83)	0.26 (0.05-1.28)	1.18 (0.35-3.94)	0.89 (0.15-5.15)	0.34 (0.07-1.68)	0.70 (0.32-1.55)	0.29 (0.06-1.46)
Living arrangement								
alone								
together	0.37 (0.15-0.94)*	/#	0.72 (0.29-1.80)	0.74 (0.31-1.75)	/#	0.57 (0.22-1.44)	0.61 (0.32-1.14)	0.66 (0.22-1.99)
institution	1.22 (0.20-7.60)	/#	1.58 (0.31-8.05)	1.15 (0.24-5.57)	/#	0.38 (0.40-3.59)	0.18 (0.03-1.03)*	1.25 (0.12-12.76)
		**Orthopedic diseases - lower extremities**	**General Arthrosis**		**Arthrosis - upper extremities **		**Arthrosis - lower extremities **	
Age		1.02 (0.98-1.07)	1.02 (0.98-1.06)		1.04 (0.99-1.10)		1.03 (0.99-1.07)	
Sex								
male								
female		1.94 (0.93-4.03)	1.50 (0.79-2.83)		2.60 (0.96-7.03)		1.77 (0.91-3.45)	
BMI		1.09 (1.02-1.16)*	1.03 (0.98-1.09)		0.95 (0.87-1.03)		1.03 (0.97-1.09)	
MoCA		0.99 (0.89-1.09)	1.00 (0.91-1.10)		0.92 (0.81-1.05)		1.04 (0.95-1.15)	
FES-I		0.91 (0.82-1.01)	1.05 (0.97-1.13)		0.97 (0.87-1.08)		1.02 (0.95-1.11)	
Education								
low								
medium		0.89 (0.40-1.96)	0.39 (0.19-0.82)*		0.61 (0.20-1.86)		0.48 (0.23-1.02)	
high		0.42 (0.15-1.20)	0.39 (0.17-0.90)*		0.72 ( 0.21-2.52)		0.33 (0.13-0.82)*	
Living arrangement								
alone								
together		1.06 (0.51-2.22)	0.97 (0.50-1.88)		/#		0.96 (0.49-1.89)	
institution		0.63 (0.10-3.83)	0.63 (0.14-2.75)		/#		0.57 (0.12-2.63)	

If not otherwise stated data is presented in Odds Ratio (OR) and 95%-Confidence Interval (CI), *p <0.05, # living situation was excluded from the model due to sparse data in the institutionalised group, which led to unstable estimates; *MoCA* Montreal Cognitive Assessment, *FES-I* Falls Efficacy Scale-International; Parkinson’s disease was excluded from analysis due to insufficient case numbers

For heart diseases, lower agreement was associated with a higher FES-I score (OR: 1.14, 95% CI 1.05–1.25) and medium education. Participants with a medium education had a 3.37 (95% CI 1.47, 7.94) times higher likelihood of inaccurate self-report as those with lower education. Reduced agreement between self-reported and GP-reported PAD was associated with increasing age (OR, 1.05; 95% CI 1.00-1.10). The likelihood of inaccurate self-reported diagnoses compared to GP-reported diagnoses of stroke increased 1.10 (95% CI (1.03, 1.18) times with every additional year of life. Agreement on cancer was associated with increasing age and increasing MoCA scores. With each additional year, the likelihood of inaccurate self-reported diagnoses compared to GP-reported cancer-diagnoses increased by 1.11 times (95% CI 1.01, 1.21) and with each additional point on the MoCA score, the likelihood increased by 1.29 (95% CI 1.01, 1.64) times. Agreement on gastrointestinal was significantly associated with increasing BMI (OR: 1.08; 95% CI 1.02–1.15) times with every additional point on the BMI scale. Agreement on renal diseases was significantly associated with increasing age and medium education. The likelihood of inaccurate self-reported renal diseases compared to the GP-reported renal diseases increased by 1.08 (95% CI 1.02, 1.14) times with every additional year of age and was 1.51 times (95% CI 0.54, 4.20) higher for participants with a medium education level compared to participants that possessed a low education level. The likelihood of inaccurately reported diagnoses of diabetes mellitus increased by 1.12 (95% CI 1.02, 1.24) times with every additional point on the FES-I score. Inaccurate self-reporting of polyneuropathy was significantly associated with increasing BMI (OR: 1.13; 95% CI 1.04–1.23). Agreement on gait disorders and frailty was significantly associated with increasing age. With every additional year, the likelihood of inaccurate self-reported gait disorders increased by 1.18 (95% CI 1.08, 1.29) times. The likelihood of inaccurate self-reported frailty increased by 1.18 (95% CI 1.09, 1.27) times. The likelihood of inaccurate self-report on the risk of falling was significantly associated with participants living in an institution. Compared to participants living alone, participants living in an institution had an 8.63 (95% CI (1.39, 53.84) times higher likelihood of inaccurate self-report compared to GP-report. Agreement on depression between self-report and GP-report was significantly associated with the FES-I. For each additional point on the FES-I score the likelihood of inaccurate self-report compared to the GP-report increased by 1.17 (95% CI 1.06, 1.29) times. Agreement on vertigo between self-report and GP-report was significantly associated with the FES-I (OR: 1.09; 95% CI 1.00–1.19) and medium education. Participants with a medium education had a 2.69 (95% CI (1.05, 6.90) times higher likelihood of inaccurate self-reported vertigo compared to GP-reported vertigo as those with lower education. The likelihood of inaccurate reporting of osteoporosis was 3.65 (95% CI (1.15, 11.57) times higher for female participants than for male participants. Agreement on general orthopaedic diseases between self-reported and GP-reported diagnoses was significantly associated with the BMI of the participants (OR:1.09; 95% CI 1.03–1.16). Similar results were found for orthopaedic diseases of the lower extremities (OR:1.03; 95% CI 1.02–1.16).

## Discussion

In the analysed population of older individuals presenting to the ED after a severe fall, the agreement between self-reported and GP-reported diagnoses was generally poor, as indicated by Cohen’s Kappa values. However, agreement varied by diagnostic group with PD and diabetes mellitus showing relatively high concordance, while conditions like arthrosis of the lower extremities and gait disorders had no agreement. Several factors were associated with a higher risk of disagreement across diagnoses, with concerns about falling being the only factor linked statistically significantly to disagreement across all conditions.

The heterogeneous results regarding agreement between self-reports and GP-reports align with previous studies in different populations of older adults [7, 17]. Higher agreement for conditions such as diabetes mellitus may be due to the significant impact these diseases have on daily life, which likely enhances the accuracy of self-reports [21]. PD often presents with motor symptoms like tremor or bradykinesia, which severely affect activities of daily living and require personalised treatment plans [22]. Similarly, diabetes mellitus is often managed through Disease Management Programmes in Germany, involving regular GP visits [7], which may explain the high agreement in diabetes mellitus reporting observed in our and previous studies [6, 7, 17, 21]. Cancer, known for its substantial impact on individuals but also their families [23], is also reported with high accuracy. Therefore, these conditions can typically be reliably self-reported, even after a severe fall, potentially reducing the need for GP verification in these cases. Nevertheless, further research is needed to confirm these findings, specifically across different countries where variations in healthcare systems and aftercare may impact the level of agreement between self-reports and GP-reports.

Moderate agreement between self-reported and GP-reported diagnoses was observed for heart diseases and stroke, consistent with previous studies [7, 17]. For instance, Hansen and colleagues analysed 3,189 participants aged 65–85 years and reported similar levels of agreement [17]. In contrast to our findings of moderate agreement on lung diseases, Hansen and colleagues found good agreement for asthma and COPD [17]. However, mixed results have been reported elsewhere. Steinkirchner and colleagues, in a study of 589 participants aged 70–95 years, found moderate agreement for asthma but poor agreement for COPD/chronic bronchitis when comparing self-reported and GP-reported diagnoses [7]. While we did not differentiate between asthma and COPD as diagnostic groups but analysed lung diseases together, this could be another possibility as to why there was found a moderate agreement for this diagnostic group. One possible reason for a low level of agreement on asthma, as suggested by Halm and colleagues in their analysis of 198 participants, is that patients with asthma often may not consider themselves to have the condition when they are asymptomatic [24]. This reinforces the idea that variations in the severity of symptoms could contribute to limited agreement [7, 24] and highlight the need for specific inquiries about diseases with fluctuating symptoms. Additionally, seeking GP input on such conditions may improve diagnostic accuracy.

Along with this assumption we found fair agreement for osteoporosis, polyneuropathy, gastrointestinal diseases and tremor, while only poor agreement was detected for PAD, depression, pain, ophthalmic diseases, incontinence, cognitive disorders, vertigo, renal diseases, sleep disorders, orthopedic diseases of the upper and lower extremities and arthrosis of the upper extremities. No agreement was detected for arthrosis of the lower extremities and gait disorders. Like Steinkirchner and colleagues, we found poor agreement between self-reported and GP-reported diagnoses for renal diseases and musculoskeletal diseases [7]. Likewise, vertigo and neuropathies showed low agreement in our study, consistent with findings from Hansen and colleagues [17]. Conditions such as neuropathies and vertigo, which often present with broad and nonspecific symptoms, may have diverse etiologies and can be assessed using different diagnostic criteria [25–27]. This variability may contribute to greater discordance between self-reports and GP-reports. Mannion et al., who compared self-reported conditions with pharmacy claims, also observed low agreement for incontinence and emotional disorders such as depression, a finding mirrored in our results [6]. Lower agreement for conditions may stem from less clear diagnostic criteria [28]. Additionally, diseases that do not require frequent monitoring or significantly impact daily life tend to show lower agreement between self-reports and medical records [29]. Conditions such as osteoporosis or PAD, which showed fair to poor agreement in our study, often remain asymptomatic for a long time [30, 31]. This may further explain the high level of discordance observed, as these diseases may not significantly affect activities of daily life, leading individuals to overlook them in self-reports. The complexity of symptoms, the lack of need for regular monitoring or treatment, and the asymptomatic nature of certain conditions suggest that patient education and communication by GPs should be intensified to improve self-reporting accuracy. To streamline the collection of pre-existing diagnoses during multifactorial assessments after severe falls, providing GPs with a predefined list of conditions – such as those mentioned above – could accelerate the process and reduce the burden on healthcare providers.

Higher concerns about falling were a relevant predictor of greater disagreement between self-reported and GP-reported diagnoses. However, no previous studies have specifically examined the possibility of temporary memory loss and the subsequent reduction in self-report accuracy regarding diagnostic information following a fall event. This finding may be particularly relevant to the present study population, as individuals with elevated FES-I scores may focus primarily on the recent fall, potentially neglecting to recall preexisting conditions. One possible explanating is Chung and colleagues’ finding that a notable minority of individuals develop post-traumatic stress disorder (PTSD) following a fall [32], a condition associated with alterations in memory and concentration due to trauma-induced stress [33]. Thus, an elevated FES-I score may be a marker of an increased stress level in some individuals, potentially leading to reduced accuracy in self-reports of preexisting diagnoses.

Another potential factor is mild cognitive impairment, which has been identified as a predictive factor for concerns about falling [34] and may reduce self-report reliability. Prior research has shown low concordance between self-reported and clinically diagnosed dementia in individuals with cognitive impairment [35]. However, in our analysis, MoCA scores were not significantly associated with disconcordance. This may be due to a stronger influence of FES-I or the mitigating effect of proxy assistance in providing medical history, potentially limiting the impact of cognitive status. Giving these findings, involving GPs in multifactorial fall risk assessments may be particularly important in individuals with high FES-I scores. Establishing FES-I cut-off values to identify thresholds at which GP involvement improves diagnostic accuracy could enhance clinical practice. Further research is warranted to validate this approach.

In this analysis, increasing age was identified as a predictive factor for a greater disagreement on PAD, cancer, renal diseases, gait disorders and frailty between self-report and GP-report. Previous studies have shown that older adults are more likely to have discrepancies regarding conditions such as myocardial infarction and stroke [7], and various heart diseases like hypertension and angina [6, 21]. Age-related cognitive decline may explain some of these discrepancies, as the risk of cognitive impairment rises with age [36, 37]. Additionally, the prevalence of multimorbidity increases with age [38], leading to a greater information burden and difficulty in recalling multiple diagnoses. Based on these findings, age should be considered a key factor, along with the short FES-I, in identifying patients who may require diagnostic support from their GP. Although improved data exchange via electronic health records may reduce this need in the future, GPs currently serve as the main point of diagnostic integration across healthcare settings. Incorporating age as one of several factors may help identify individuals for whom GP input would enhance diagnostic accuracy. Future research is needed to establish clear thresholds for when such support is warranted.

The higher risk of disagreement in women diagnosed with osteoporosis, compared to men, aligns with findings from Mannion et al., who reported a 5 times higher likelihood of discordance for osteoporosis [6]. Osteoporosis is often asymptomatic until fractures occur and women are more frequently affected [39]. A study by Lewiecki et al. found that women often underestimate the severity of osteoporosis [39], despite its relevance to fall-related fractures [40]. Therefore, this diagnosis requires particular attention, and a specific question should be included in the risk assessment. Interestingly, higher MoCA scores were associated with disagreement on cancer diagnoses. Although a higher score indicates better cognitive function [10], this discordance suggests that even cognitively intact individuals may provide incomplete diagnostic information. Thus, obtaining details from GPs is crucial not only for those with cognitive impairment.

The predominantly poor agreement between self-reported and GP-reported diagnoses underscores the importance of accurately assessing preexisting conditions as part of the multifactorial assessment following a severe fall. As there is broad consensus that improved communication between physicians and patients enhances treatment adherence [41], health-outcomes [42, 43] and that optimised information delivery improves understanding and recall of diagnoses [44, 45], strengthening doctor-patient communication may also improve the accuracy of self-reported medical histories. Enhanced communication could increase patient awareness of existing conditions, potentially improving interactions in the ED and facilitating early treatment modifications. Additionally, actively involving caregivers or proxies – particularly in high-stress environments like the ED – could provide essential diagnostic insights. However, this approach requires careful considerations of available resources.

Given the challenges in assessing preexisting conditions, digitalisation of healthcare systems is crucial [46], since assessing electronic health information in the ED has been associated with improvements in care processes [47]. In Germany, the electronic health card (eGK) has the potential to enhance clinical decision-making [48] particularly in the ED where timely access to patient history is critical. However, existing barriers, such as the coexistence of paper-based record-keeping systems and various electronic systems in the German healthcare landscape, continue to hinder the widespread implementation of information transfer via the eGK [46]. Further, a comprehensive infrastructure that enables the exchange of clinically relevant data across all healthcare providers must be established [49]. Future research should investigate the eGK as a valuable tool for accessing diagnostic data in ED settings, with proper authorisation protocols in place and full compliance with data protection regulations, including personal authorisation and consent from the insured individual [50].

### Limitations and strengths

Unlike other studies that have examined a comparison of concordance of diagnostic information, this analysis is the first to investigate this issue specifically in older adults visiting the ED after a severe fall. The study directly compared participants’ self-reports with the GP-reports, with the GP-report being treated as the gold standard. However, it is important to acknowledge that GPs may not always serve as the definitive source of diagnostic information. GPs may lack diagnostic data collected by various specialists that was not transferred to them, an issue assumed in previous research [7]. In our study GPs reported more diseases apart from ophthalmic diseases which have been detected as the only diagnostic group that were reported more frequently by participants than by their GPs. One possible hypothesis may be that even small losses in visual acuity can be perceived as a major subjective impairment which could justify a higher frequency in the self-reports, but may also indicate a lack of communication between specialists and GPs. Additionally, this analysis included only participants whose GPs provided diagnostic information, potentially introducing a selection bias. GPs who participated in the study by sending diagnostic data may have greater interest in detailed documentation. Diagnoses such as frailty or gait disorders, which are often documented by GPs, may not be perceived by participants as formal diagnoses, but rather as everyday limitations, and therefore may not be reported. Similarly, cancer diagnoses were included regardless of time since diagnosis, remission status, or whether the condition was resolved and no longer impacting daily life. This may partly explain discrepancies between self-reported and GP-reported diagnoses. While such conditions are important due to their implications for fall risk and functional decline [51], clear communication between GPs and patients remains essential to ensure awareness and understanding of these diagnoses. This discrepancy could explain the high disconcordance between self-reports and GP-reports for these conditions. Although diagnoses like frailty are essential to examine, as they increase the risk of future functional decline and falls [51], communication between GPs and patients is particulary important in ensuring patients are informed about such diagnoses. Improved dialogue may help bridge the gap in understanding and recognition of these critical risk factors. Living situation was initially included as an independent variable (categorised as living alone, with others, or institutionalised). However, due to the small number of institutionalised participants, several models produced highly unstable or implausible odds ratios, likely driven by sparse data. As a result, living situation was excluded from the final models for overall diagnoses, cancer, cognitive impairment, sleep disorders, tremor/RLS, and arthrosis of the upper extremities. Future studies should include larger institutionalised subgroups, based on a priori power calculations, to more robustly investigate the potential influence of living situation across a bigger sample.

## Conclusion

Participants appeared to be more aware of diagnoses that require frequent monitoring or are associated with persistent symptoms, such as diabetes mellitus. In contrast, conditions that are often asymptomatic, such as osteoporosis, may require more focused inquiry during assessments.

Based on predictive factors identified in this analysis – such as age, sex, higher FES-I scores and MOCA values – special attention should be given to individuals with these characteristics during post-fall evaluations. For individuals with this risk profile, contacting the GP to obtain information on preexisting conditions may be beneficial, enabling more tailored aftercare. This could aid in both falls prevention and recovery following a fall.

To improve patient understanding and recall of diagnoses, GP communication should be tailored to meet the specific needs of older individuals, as this may enhance concordance between self-reported and GP-reported diagnostic information. Future research should focus on incorporating multiple sources of diagnostic data to optimise information retrieval and improve the accuracy of assessing preexisting conditions. Increased digitalisation in the healthcare system could play a key role in supporting both future research and patient care, facilitating more efficient access to comprehensive medical records and improving clinical decision-making.

## Supplementary Information

Below is the link to the electronic supplementary material.

Supplementary Material 1

## Data Availability

The data that support the findings of this study are not openly available due to reasons of sensitivity and are available from the corresponding author upon reasonable request. Data are located in controlled access data storage at University of Oldenburg.

## Abbreviations

CI: confidence interval
BMI: body mass index
ED: emergency department
FES-I: Falls Efficacy Scale-International
GP: general practitioner
ICD-10: International Classification of Disease 10
MoCA: Montreal Cognitive Assessment
OR: Odd’s Ratio
PAD: peripheral arterial disease
PD: Parkinson’s Disease
SD: standard deviation
SeFallED: Sentinel Fall presenting to the Emergency Department
VIFs: Variance Inflation Factors
