# Supplementary material for: Emergency department visits due to severe falls: comparing patient self- reports and general practitioner records: A cross-sectional study
Source: BMC Geriatr. 2025 Oct 6;25:757. doi: 10.1186/s12877-025-06411-9 (PMC12502360; doi:10.1186/s12877-025-06411-9)
Supplement: Supplementary file 1 — Supplementary Material 1 [file 12877_2025_6411_MOESM1_ESM.docx]

| **Supplementary Table 1**. Diagnostic groups and included ICD10-codes | |
| --- | --- |
| *Diagnostic groups* | *Included ICD10-codes* |
| Heart diseases | I10-I15, I20-I25, I47.-, I48.-, I49.-, R00.- I44.- I45.-, I50.-, Z45.-, Z95.- |
| PAD (Peripheral Arterial Disease) | I70.- |
| Stroke (apoplexy) | I63.-, I64.-, I65.-, I67.-, I69.-, G45.- |
| Cancer | C00.- - C80.- |
| Lung diseases | J40.- - J47.- |
| Gastrointestinal diseases | K21.-, K22.-, K29.- K56.-, K57.- K58.-, K59.-, K80.-, K83.-, R13.- |
| Renal diseases | N18.- N19.-, N28.- |
| Incontinence | R15.- R32.-, R39.- |
| Ophthalmic diseases | H18.- H25.-, H26.-, H33.-, H35.-, H40.-, H47.-, H52.- H53.-, Z96.- |
| Diabetes mellitus | E10.- - E14.- |
| Polyneuropathy | G60.- - G64.- |
| Cognitive impairment | F70-. – F73.-, , F00.-* - F03.-, G30.- +,R41.- |
| PD (Parkinson`s disease) | G20.- - G22* |
| Gait disorders | R26.- |
| Frailty | R54 |
| Risk of falling | R29,6 |
| Depression | F32.-, F33.-, F45.-, R53.- |
| Sleeping disorders | G47.- |
| Pain | F45.-, R52.-, M79.- |
| Vertigo | H81.- R42.-, R55.- |
| Tremor | G25.- R25.- Restless Legs Syndrome |
| Osteoporosis | M80.- - M82.-* |
| Orthopaedic diseases (both upper and lower  Extremities) | M21.-, M23.-, M43.-, M47.-, M48.-, M50.- - M54.-, M75.-, M94.-, Q66.-, Z96.- |
| Arthrosis (both upper and lower extremities) | M15.- - M19.- |
